# Association between serotonin 2A receptor (HTR2A) genetic variations and risk of hypertension in a community-based cohort study

**DOI:** 10.1186/s12881-019-0927-3

**Published:** 2020-01-06

**Authors:** Jung Ran Choi, Minhee Jeon, Sang Baek Koh

**Affiliations:** 1 Institute of Genomic Cohort, Yonsei University Wonju College of Medicine, Wonju, Republic of Korea; 2 Department of Preventive Medicine, Yonsei University Wonju College of Medicine, Wonju, Republic of Korea

**Keywords:** Hypertension, Serotonin 2A receptor gene, Cross sectional study, Genotype

## Abstract

**Background:**

Hypertension is one of the risk factors for obesity-related cardiovascular diseases. We investigated whether genetic variations in serotonin 2A receptor (*HTR2A*) were associated with hypertension.

**Methods:**

We carried out a cross-sectional study in cohorts A (Ansan-Ansung cohort, *N* = 6039) and B (Wonju-Pyengchang cohort, *N* = 7524). Several genetic variants in HTR2A including rs7330636, rs9590999, rs2183057, and rs4942595 were selected and genotyped.

**Results:**

In hypertensive participants in cohort A, the baseline systolic blood pressure and body mass index were 141.80 ± 17.20 mg/dL and 24.48 ± 4.75 kg/m^2^, respectively, which were higher than in those without hypertension (*p* < 0.001). rs4942595TC genotype was associated with hypertension in cohort A (OR = 0.739), after adjusting for variables. Subjects with rs4942578AA genotype had a decreased risk of hypertension after adjusting for clinical factor (OR = 0.735) in cohort B, and an elevated risk of hypertension in cohort A (OR = 1.562). The logistic regression analysis showed that participants with rs4941573TC genotype were 1.327 times more likely to have a higher blood pressure than those with TT genotype (95% CI 1.101–1.599) in cohort B. Whereas, the OR for developing hypertension in subjects with rs17069883CC genotype compared to those with AA genotype was 1.447 (95% CI 1.018–2.056; *p* for trend = 0.040) in cohort A.

**Conclusions:**

*HTR2A* genetic variations were associated with hypertension risk in our study.

## Background

Hypertension is considered not only a risk factor contributing to cardiovascular disease, but also a health issue in the community [1, 2]. The known risk factors for hypertension are age, low physical fitness, tobacco usage, unhealthy eating, and high salt consumption [1]. Hypertension is a complex disease affected by genes, environmental factors, and their interactions [3, 4].

Twin and family studies have shown that generally 20–60% of blood pressure variations could be ascribed to genetics [3, 5]. Until now, many candidate genes have been shown to be involved in the regulation of blood pressure and susceptibility of hypertension [3, 6, 7]. Moreover, several genome-wide association studies demonstrated multiple susceptibility loci, associated with hypertension and blood pressure [8, 9].

Among the physiological factors for regulating blood pressure, a neurotransmitter and hormone serotonin (5-hydroxytryptamine, 5-HT) were considered. Its precise function in normal or altered blood pressure is unclear, likely because of the complex events of blood 5-HT in vitro [10, 11]. The association of serotonin and development of hypertension was investigated by the blood platelets, which served as a primary storage location, and were easily accessible source of serotonin [10]. Blood platelet serotonin 2A receptor (*HTR2A*, also known as 5-HTR2A), regulates platelet activation and serotonin release; whereas 5-HTR2A located in vascular smooth muscle, endothelial cells, and cardiomyocytes regulate blood pressure and heart rate [12]. *HTR2A* encodes the 5HT2a receptor, which is expressed in the peripheral vasculature, and is involved in hypertension due to its vasoconstrictive effect [3, 13].

Recent studies suggest that genetic variants in the *HTR2A* gene may be associated with hypertension [14], and abdominal obesity [15, 16]. However, no associations were described between *HTR2A* genetic variations and hypertension in smaller case control studies in Chinese or Japanese subjects [17, 18]. We investigated whether genetic variations of *HTR2A* were associated with the risk of hypertension in two different community-based cohorts.

## Methods

### Study population

**Fig. 1:**
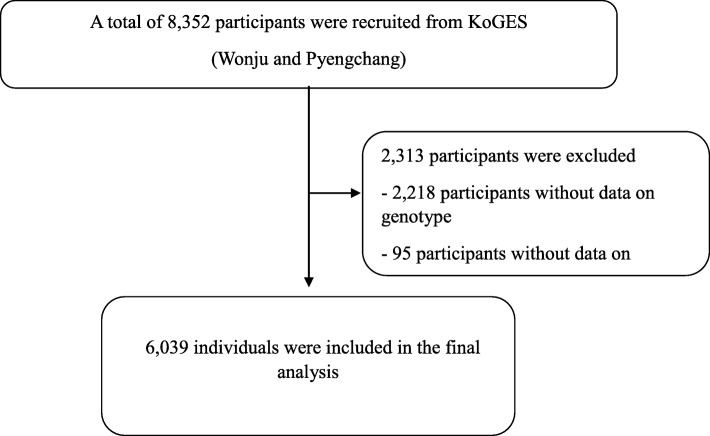
Flowchart showing the final selection in cohort A. KOGES, The Korean Genome and Epidemiology study

**Fig. 2:**
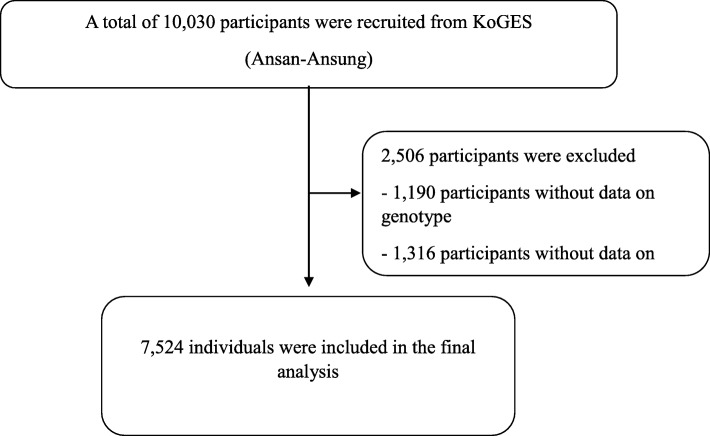
Flowchart showing the final selection in cohort B. KOGES, The Korean Genome and Epidemiology study

This study was performed with individuals from a community-based cohort (the Ansan-Ansung and Wonju-Pyengchang cohort) within the Korean Genome Epidemiology Study (KoGES). The Ansan-Ansung cohort is a longitudinal survey supported by the Korean government (Korean National Research Institute of Health, Korean Centers for Disease Control and Prevention, and the Ministry of Health and Welfare), to examine the genetic and environmental factors of chronic disease in Koreans [19–21]. Korean adults was recruited from the following two sites: Ansung and Ansan, which is a rural district with roughly 190,000 and 693,000 residents, respectively [22]. A total of 10,038 adults (5018 from Ansung and 5020 from Ansan) had a baseline health examination at the Ajou University Medical Center and the Korea University Ansan Hospital from June 2001 to January 2003. Follow-up surveys were performed biennially [22]. Additionally, all adults residing in rural areas of Wonju and Pyengchang in South Korea, where demographic changes are uncommon, participated in the study [23–25]. The study protocol was approved by the Institutional Review Board of Wonju Christian Hospital and Ajou University Medical Center, and the Korea University Ansan Hospital. All participants provided written informed consent. We excluded the participants with incomplete data and without the result of sequencing in HTR2A gene (Figs. 1 and 2). In total, 6039 participants (cohort A) and 7524 (cohort B) were eligible for this study. Hypertension was defined as a systolic blood pressure (SBP) ≥ 140 mmHg, and/or diastolic blood pressure (DBP) ≥ 90 mmHg, and/or current treatment with antihypertensive medications. All the participants were examined after fasting.

### Anthropometric and laboratory measurements

Anthropometric indices including the body mass index (BMI), smoking status, regular physical exercise and several other parameters were investigated. The waist circumference was obtained using a tape measure (SECA-200, SECA, Hamburg, Germany). Systolic (SBP) and diastolic blood pressure (DBP) were measured twice by a standardized mercury sphygmomanometer (Baumanometer, Copiague, New York). Blood sample was extracted from participants after fasting. Fasting glucose was estimated by a glucose oxidase-based assay. The serum concentrations of low-density lipoprotein (LDL) cholesterol, high-density lipoprotein (HDL) cholesterol, and triglycerides (TGs) were examined using the enzymatic calorimetric method (Advia 1650, Siemens, Tarrytown, New York). Alcohol and smoking habits were identified by self-questionnaires [26].

### Sequencing and genotype

We analyzed several genetic variants in HTR2A gene. PCR primers were designed to amplify *HTR2A* fragments. PCR products were sequenced by a BigDye Terminator Cycle Sequencing Kit (version 3.1, ABI, Foster City, CA, USA), and an ABI 3730 × 1 automated sequencer (Applied Biosystems, Foster City, CA, USA). SNPs confirmed in the *HTR2A* gene were genotyped. Genomic DNA was drawn from 5 mL of peripheral venous blood using an isolation kit (QuickGene SP Kit DNA whole blood, Fujifilm, Tokyo, Japan). Genotyping was performed using the TaqMan fluorogenic 5′ nuclease assay (ABI) [27].

We evaluated the association between genetic variants in HTR2A and risk of hypertension in each cohort, however, some of them did not show the association of genetic variants of HTR2A and risk of hypertension. In cohort A and B, several single nucleotide polymorphisms (SNPs) which shown significant and non-significant associations of risk of hypertension were reported (e.g. rs7330636, rs9590999, rs2183057) in this study.

### Statistical analysis

Group differences for categorical variables were analyzed using chi-square test, and the continuous variables were estimated by Student’s *t*-test. The association between genotype and the risk of hypertension was assessed using odds ratios (ORs) and 95% confidence intervals (CIs), from chi-square tests and logistic regression analyses. The results are shown as mean ± standard error range. The *p*-values for each genotype were calculated by ANOVA test. A *p*-value with three decimal places was considered as statistically significant difference between no hypertension and hypertensive individuals. All analyses were determined using SPSS software, version 23.0 (SPSS Inc., Chicago, IL, USA). We adjusted for age, gender, smoking status, alcohol consumption and regular exercise in Model 1. Model 2 was adjusted for Model 1 and systolic blood pressure, total cholesterol and baseline body mass index, additionally.

## Results

### Characteristics of the study population according to the genotype of HTR2A

Characteristics of the population are shown in Table 1. Total 1418 subjects, who developed hypertension in cohort A, were aged 61.59 ± 6.12 years, and 46.8% (*n* = 664) of the total population were men. In the participants who developed hypertension in cohort B, the baseline SBP and fasting glucose levels were 140.06 ± 16.21 mmHg and 91.18 ± 30.71 mg/dL, respectively, which were higher than in those without hypertension (*p* < 0.001) (Table 1).

**Table 1:** Demographic characteristics of study population in cohort A and B

Cohort A (*N* = 6039)			
Variables, N (%)	No Hypertension (*N* = 4621)	Hypertension (*N* = 1418)	*p*-value
Age (y)	60.00 ± 9.53	61.59 ± 6.12	<.0001
Gender, Male (%)	1792 (38.8)	664 (46.8)	<.0001
SBP (mmHg)	116.44 ± 11.75	141.80 ± 17.20	<.0001
DBP (mmHg)	74.98 ± 7.74	89.20 ± 10.11	<.0001
BMI (kg/m^2^)	23.68 ± 3.38	24.48 ± 4.75	<.0001
WC (cm)	83.01 ± 8.68	86.16 ± 8.37	<.0001
Fasting glucose (mg/dL)	96.93 ± 21.69	113.22 ± 42.24	<.0001
TC (mg/dL)	197.73 ± 36.53	204.76 ± 41.73	<.0001
TG (mg/dL)	140.70 ± 89.82	178.30 ± 135.51	<.0001
HDL (mg/dL)	45.38 ± 10.97	45.78 ± 11.70	0.247
Current smoker, n (%)	807 (17.5)	219 (15.4)	0.158
Alcohol consumer, n (%)	1977 (42.8)	563 (39.7)	< 0.001
Regular Exercise, n (%)	1179 (25.5)	345 (24.3)	0.017
Cohort B (*N* = 7524)			
Variables, N (%)	No Hypertension (*N* = 4999)	Hypertension (*N* = 2525)	*p*-value
Age (y)	50.42 ± 8.40	55.36 ± 8.65	<.0001
Gender, Male (%)	2325 (46.50)	1257 (49.78)	0.007
SBP (mmHg)	112.70 ± 11.57	140.06 ± 16.21	<.0001
DBP (mmHg)	74.80 ± 7.80	92.05 ± 9.23	<.0001
BMI (kg/m^2^)	19.56 ± 9.86	18.73 ± 11.62	0.002
WC (cm)	80.92 ± 8.73	85.98 ± 8.94	<.0001
Fasting glucose (mg/dL)	88.93 ± 23.25	91.18 ± 30.71	<.0001
TC (mg/dL)	196.06 ± 35.19	204.00 ± 38.02	<.0001
TG (mg/dL)	140.93 ± 99.52	176.56 ± 127.62	<.0001
HDL (mg/dL)	49.84 ± 11.68	48.56 ± 11.81	<.0001
Current smoker, n (%)	1143 (22.86)	492 (19.48)	<.0001
Alcohol consumer, n (%)	2371 (47.42)	1192 (47.20)	0.586
Regular Exercise, n (%)	3441 (68.83)	1733 (68.63)	0.860

*BMI* body mass index, *WC* waist circumference, *SBP* systolic blood pressure, *DBP* diastolic blood pressure, *TC* total cholesterol, *TG* triglyceride, *HDL* high-density lipoprotein

The genotype frequency of the studied loci is shown in Table 2. Genotype frequencies for all SNPs were in accordance with the Hardy-Weinberg equilibrium. Distribution of *HTR2A* rs2183057 genotype frequencies differed significantly between patients with or without hypertension. We observed a statistically significant increase in rs17069883CC genotype frequency in the group of patients with hypertension (23.3 vs. 19.3% in no hypertension group, *p* = 0.041), and a decrease in the number of rs4942595TC genotype heterozygotes in no hypertension group (47.1 vs. 53.1%, *p* = 0.041) (Table 2).

**Table 2:** Distribution of *HTR2A* genetic variations according to development of hypertension

SNP	Genotype	No Hypertension (*N* = 4621), N (%)	Hypertension (*N* = 1418), N (%)	*p*-value
Cohort A (*N* = 6039)				
rs7330636	GG	555 (85.9)	989 (89.2)	0.092
	GA	89 (13.8)	115 (10.4)	
	AA	2 (0.3)	5 (0.5)	
rs9590999	CC	167 (25.1)	335 (29.2)	0.056
	CT	354 (53.2)	545 (47.6)	
	TT	144 (21.7)	266 (23.2)	
rs2183057	AA	253 (38.0)	401 (34.9)	0.026
	AG	331 (49.7)	553 (48.1)	
	GG	82 (12.3)	195 (17.0)	
rs4942595	TT	168 (25.2)	339 (29.5)	0.041
	TC	354 (53.1)	541 (47.1)	
	CC	145 (21.7)	269 (23.4)	
rs4531630	AA	168 (25.3)	338 (29.6)	0.062
	AG	344 (51.7)	584 (51.1)	
	GG	153 (23.0)	221 (12.2)	
rs17069883	AA	197 (29.5)	287 (25.0)	0.041
	AC	341 (51.1)	594 (51.7)	
	CC	129 (19.3)	268 (23.3)	
rs4942578	CC	258 (38.7)	431 (37.5)	0.225
	CA	320 (48.0)	530 (46.1)	
	AA	89 (13.3)	188 (16.4)	
Cohort B (*N* = 7524)				
rs4942578	CC	1834 (39.2)	949 (40.2)	0.605
	CA	2171 (46.5)	1089 (46.2)	
	AA	668 (14.3)	320 (13.6)	
rs985933	AA	1526 (32.8)	781 (33.2)	0.496
	AG	2257 (48.5)	1154 (49.1)	
	GG	875 (18.8)	414 (17.6)	
rs4941573	TT	1184 (25.4)	565 (24.0)	0.196
	TC	2314 (49.6)	1221 (51.8)	
	CC	1172 (25.1)	571 (24.2)	
rs6313	GG	1229 (26.3)	584 (24.8)	0.153
	GA	2301 (49.2)	1218 (51.7)	
	AA	1143 (24.5)	556 (23.6)	
rs1328685	TT	4031 (86.3)	2054 (87.1)	0.259
	TC	611 (13.1)	295 (12.5)	
	CC	31 (0.7)	9 (0.4)	
rs2567675	TT	2823 (60.4)	1483 (62.9)	0.124
	TC	1616 (34.6)	762 (32.3)	
	CC	234 (5.0)	112 (4.8)	

The analysis of the data showed that carriers of rs7330636GA genotype had a lower risk of hypertension (OR = 0.644, 95% CI 0.437–0.949). The rs9590999CT genotype was associated with a decreased risk of hypertension (OR = 0.738 95% CI 0.551–0.989) in cohort A.

Subjects with rs4942578AA genotype had a decreased risk of hypertension after adjusting for clinical factor such as age and gender (OR = 0.735, *p* = 0.010) in cohort B, while, those with rs4942578AA genotype had an elevated risk of hypertension in cohort A (OR = 1.562, *p* = 0.020). The logistic regression analysis showed that participants with rs6313GA genotype were 1.365 times more likely to have a higher blood pressure than those with GG genotype (OR = 1.365; 95% CI 1.135–1.643; *p* = 0.003) in cohort B. Meanwhile, the odds ratio for developing hypertension in subjects with rs4941573TC genotype compared to those with TT genotype was 1.327 (95% CI 1.101–1.599; *p* for trend = 0.003) in cohort B (Table 3).

**Table 3:** Association of genetic polymorphisms of *HTR2A* gene and risk of hypertension

SNP		Crude	*p*	Model 1	*p*	Model 2	*p*
Cohort A							
rs7330636	GG	1.000		1.000		1.000	
	GA	0.725 (0.540–0.974)	0.033	0.725 (0.538–0.976)	0.034	0.644 (0.437–0.949)	0.026
	AA	1.403 (0.271–7.255)	0.686	1.520 (0.292–7.922)	0.619	1.415 (0.206–9.735))	0.724
rs9590999	CC	1.000		1.000		1.000	
	CT	0.767 (0.610–0.965)	0.023	0.757 (0.601–0.953)	0.018	0.738 (0.551–0.989)	0.042
	TT	0.921 (0.700–1.212	0.921	0.914 (0.692–1.205)	0.522	0.880 (0.624–1.239)	0.463
rs2183057	AA	1.000		1.000		1.000	
	AG	1.054 (0.856–1.298)	0.620	1.067 (0.856–1.316)	0.546	1.131 (0.865–1.479)	0.369
	GG	1.500 (1.109–2.030)	0.009	1.500 (1.106–2.034)	0.009	1.448 (0.990–2.118)	0.056
rs4942595	TT	1.000		1.000		1.000	
	TC	0.757 (0.603–0.952)	0.017	0.748 (0.594–0.941)	0.013	0.739 (0.552–0.989)	0.042
	CC	0.919 (0.699–1.209)	0.547	0.911 (0.692–1.201)	0.510	0.907 (0.645–1.276)	0.574
rs4531630	AA	1.000		1.000		1.000	
	AG	0.844 (0.672–1.060)	0.144	0.834 (0.663–1.050)	0.122	0.792 (0.592–1.059)	0.115
	GG	0.718 (0.544–0.947)	0.019	0.705 (0.533–0.933)	0.015	0.658 (0.462–0.938)	0.021
rs17069883	AA	1.000		1.000		1.000	
	AC	1.196 (0.955–1.497)	0.119	1.197 (0.954–1.502)	0.121	1.197 (0.896–1.598)	0.224
	CC	1.426 (1.080–1.882)	0.012	1.449 (1.095–1.916)	0.009	1.447 (1.018–2.056)	0.040
rs4942578	CC	1.000		1.000		1.000	
	CA	0.991 (0.806–1.220)	0.935	1.007 (0.816–1.241)	0.950	1.054 (0.808–1.376)	0.696
	AA	1.264 (0.941–1.699)	0.120	1.288 (0.956–1.736)	0.096	1.562 (1.072–2.275)	0.020
Cohort B							
rs4942578	CC	1.000		1.000		1.000	
	CA	0.969 (0.871–1.079)	0.569	0.979 (0.876–1.094)	0.710	0.894 (0.759–1.053)	0.180
	AA	0.926 (0.793–1.081)	0.328	0.949 (0.808–1.114)	0.521	0.735 (0.582–0.929)	0.010
rs985933	AA	1.000		1.000		1.000	
	AG	0.999 (0.893–1.117)	0.986	1.013 (0.902–1.138)	0.824	1.057 (0.892–1.252)	0.521
	GG	0.924 (0.799–1.069)	0.289	0.922 (0.793–1.073)	0.293	0.789 (0.631–0.986)	0.037
rs4941573	TT	1.000		1.000		1.000	
	TC	1.106 (0.979–1.249)	0.106	1.137 (1.001–1.290)	0.048	1.327 (1.101–1.599)	0.003
	CC	1.021 (0.886–1.176)	0.774	1.033 (0.891–1.196)	0.670	1.113 (0.896–1.383)	0.332
rs6313	GG	1.000		1.000		1.000	
	GA	1.114 (0.987–1.257)	0.079	1.157 (1.021–1.311)	0.023	1.365 (1.135–1.643)	0.001
	AA	1.024 (0.889–1.179)	0.745	1.042 (0.899–1.206)	0.586	1.130 (0.911–1.403)	0.267
rs1328685	TT	1.000		1.000		1.000	
	TC	0.948 (0.817–1.100)	0.478	0.939 (0.805–1.096)	0.427	0.982 (0.786–1.228)	0.875
	CC	0.570 (0.271–1.199)	0.138	0.544 (0.252–1.175)	0.121	0.292 (0.097–0.883)	0.029
rs2567675	TT	1.000		1.000		1.000	
	TC	0.898 (0.807–0.999)	0.047	0.868 (0.776–0.969)	0.012	0.832 (0.708–0.979)	0.027
	CC	0.911 (0.721–1.151)	0.435	0.880 (0.690–1.123)	0.305	0.799 (0.561–1.138)	0.214

Model 1 was adjusted for gender, smoking status, alcohol consumption, regular exercise and age. Model 2 was adjusted for Model 1 and systolic blood pressure, total cholesterol and baseline body mass index additionally

Whereas, rs1360020 and rs4941573 were not associated with an elevated risk of hypertension in cohort A (OR = 1.294 95% CI 0.908–1.843; OR = 1.218 95% CI 0.853–1.740) (Additional file 1: Table S2). Similarly, in cohort B, rs9316232 and rs1360020 were not associated with hypertension (*p* = 0.644 vs. *p* = 0.109) (Additional file 1: Table S3). We added adjusted *p* value and 95% confidential interval using multiple testing in Additional file 1: Tables S4, S5 and S6.

## Discussion

Genetic variations in the serotonin 2A receptor gene were found to be associated with hypertension, in a community-based cohort study. Heterozygosity of T102C (rs6313) was associated with the blood pressure of 140/95 mmHg or higher, after adjustment for demographic covariates, such as age, gender and BMI. We identified an association between rs4942578 and rs6313, located in intron, and an elevated risk of hypertension.

Even though the mechanism of these connection exists uncertain, there is sufficient evidence available ascertaining that serotonin and serotonergic neurotransmission play a crucial role in cardiovascular regulation. Serotonin is a vasoactive monoamine, having amphibolic features demonstrated by its capability to induce either vasoconstriction and blood pressure enhancement [16, 28]. The human brain serotonergic inductivity in the hypothalamic-pituitary axis has been founded to increase with a decrease in resting blood pressure, and vice versa [16]. The blood pressure increasing effects of serotonin seem to be regulated by 5-HT_2A_ receptor, while 5-HT_1A_ receptor activation leads to sympathoinhibition and vasodilation [16]. Therefore, the *HTR2A* polymorphisms might play a role in the blood pressure regulation by serotonin, most likely by central mechanisms [14, 16] and recent studies suggested that polymorphic variations in *HTR2A* gene may be associated with hypertension [14].

Association between *HTR2A* SNPs and hypertension has been demonstrated in different ethnic groups [3, 18, 29, 30]. SNPs in several other genes have also been shown to be associated with hypertension such as A1161 polymorphism in angiotensin II type I receptor gene (*AGTR1*) and *CYP11B2* gene polymorphism [14, 31–33]. The ß2 adrenergic receptor has been involved in the cause of hypertension owing to its role in vasodilatation, and a significant association was verified between the genetic polymorphism of ß2 adrenergic receptor and hypertension [14]. A meta-analysis has shown that *RGS2* 1891-1892del TC polymorphism and *CYP4A11* T8590C polymorphism were associated with hypertension risk [3]. Also, the T58C promoter polymorphism of the bradykinin ß2 receptor gene was associated with hypertension in a Japanese population [14, 34]. Halder I et al. suggested that elevated blood pressure was associated with both -1438GG and 2416TT genotypes in *HTR2A* gene [16]. Similarly, we identified significant associations between genetic variants in *HTR2A* and risk of hypertension.

There are few strengths and limitations of our study. *HTR2A* gene polymorphisms were evaluated in two larger community-based cohorts compared to earlier studies, thus, the sample size was large enough to reach adequate statistical power. Moreover, association of *HTR2A* rs4942578 and rs4941573 polymorphisms with hypertension had not been investigated before. However, our findings may not be applicable to other populations, especially different ethnicities or with younger age. Additionally, we did not find sufficient studies to corroborate the association between *HTR2A* gene polymorphisms and the risk of hypertension found in this community-based cohort study. In cohort A, basic characteristics of populations included in the analyses and those excluded from the analyses were significantly different including age, gender, SBP, DBP, BMI, glucose, smoker and exercise and this might lead to selection bias.

## Conclusions

We identified significant associations between several *HTR2A* SNPs and the risk of hypertension. Further studies are required to elucidate the in vivo effects and identify functional loci in order to explain a mechanistic basis for observed associations. Confirmatory studies are also warranted using larger sample size and definitive genetic methodologies, such as family-based designs or populations with genetic stratification, along with application of genomic control procedures.

## Supplementary information

**Additional file 1: Table S1.** Basic characteristics of participants included and excluded. T**able S2.** Non-significant association of genetic polymorphisms of *HTR2A* gene and risk of hypertension. **Table S3.** Non-significant association of genetic polymorphisms of *HTR2A* gene and risk of hypertension. **Table S4.** Distribution of *HTR2A* genetic variations according to development of hypertension. **Table S5.** Association of genetic polymorphisms of *HTR2A* gene and risk of hypertension. **Table S6.** Association of genetic polymorphisms of *HTR2A* gene and risk of hypertension.

## Data Availability

The datasets generated and/or analyzed during the current study are available in the the Korea National Institute of Health (KNIH) repository (http://www.nih.go.kr/NIH_NEW/main.jsp).

## Abbreviations

BMI: Body mass index
CI: Confidence interval
DBP: Diastolic blood pressure
HDL: High-density lipoprotein
*HTR2A*: Serotonin 2A receptor
KoGES-ARIRANG: Korean Genome and Epidemiology Study on the Atherosclerosis Risk of Rural Areas in the Korean General Population
OR: Odds ratio
SBP: Systolic blood pressure
TG: Triglyceride
WC: Waist circumference
