# Supplementary material for: Association between serotonin 2A receptor (HTR2A) genetic variations and risk of hypertension in a community-based cohort study
Source: BMC Med Genet. 2020 Jan 6;21:5. doi: 10.1186/s12881-019-0927-3 (PMC6943889; doi:10.1186/s12881-019-0927-3)
Supplement: Supplementary file 1 — Additional file 1: Table S1. Basic characteristics of participants included and excluded. Table S2. Non-significant association of genetic polymorphisms of HTR2A gene and risk of hypertension. Table S3. Non-significant association of genetic polymorphisms of HTR2A gene and risk of hypertension. Table S4. Distribution of HTR2A genetic variations according to development of hypertension. Table S5. Association of genetic polymorphisms of HTR2A gene and risk of hypertension. Table S6. Association of genetic polymorphisms of HTR2A gene and risk of hypertension. [file 12881_2019_927_MOESM1_ESM.docx]

Table S1. Basic characteristics of participants included and excluded.

| Cohort A |  |  |  |
| --- | --- | --- | --- |
| Variables, N (%) | Excluded (N=2313) | Included (N=6039) | p |
| Age (y) | 58.02±9.30 | 60.37±8.87 | <.0001 |
| Gender, Male (%) | 845 (36.5) | 2456 (40.7) | <.0001 |
| SBP (mmHg) | 126.71±20.17 | 122.39±17.05 | <.0001 |
| DBP (mmHg) | 79.33±12.45 | 78.32±10.31 | 0.001 |
| BMI (kg/m^2^) | 24.40±3.53 | 23.87±3.76 | <.0001 |
| WC (cm) | 84.12±8.81 | 83.73±8.71 | 0.073 |
| Fasting glucose (mg/dL) | 98.68±22.84 | 100.75±28.74 | 0.001 |
| TC (mg/dL) | 199.25±37.71 | 199.38±37.93 | 0.886 |
| TG (mg/dL) | 147.56±92.70 | 149.53±103.63 | 0.399 |
| HDL (mg/dL) | 45.51±11.26 | 45.48±11.14 | 0.893 |
| Current smoker, n((%) | 266 (11.5) | 1026 (17.0) | <.0001 |
| Alcohol , n (%) | 989 (42.8) | 2540 (42.1) | 0.239 |
| Exercise, n (%) | 797 (34.5) | 1524 (25.2) | <.0001 |

| Cohort B |  |  |  |
| --- | --- | --- | --- |
| Variables, N (%) | Excluded (N=2506) | Included (N=7524 | p |
| Age (y) | 52.28±8.95 | 52.29±8.92 | 0.984 |
| Gender, Male (%) | 1210 (48.3) | 3548 (47.2) | 0.169 |
| SBP (mmHg) | 122.00±18.26 | 122.34±18.93 | 0.430 |
| DBP (mmHg) | 80.78±11.56 | 80.65±11.77 | 0.628 |
| BMI (kg/m^2^) | 18.26±11.01 | 19.52±10.32 | <.0001 |
| WC (cm) | 82.61±9.53 | 82.61±9.01 | 0.992 |
| Fasting glucose (mg/dL) | 88.90±24.46 | 89.93±28.24 | 0.100 |
| TC (mg/dL) | 197.47±36.77 | 198.84±36.95 | 0.109 |
| TG (mg/dL) | 154.06±113.05 | 153.03±108.82 | 0.683 |
| HDL (mg/dL) | 49.61±11.82 | 49.47±11.89 | 0.623 |
| Current smoker, n((%) | 582 (23.25) | 1673 (22.2) | <.0001 |
| Alcohol , n (%) | 1210 (48.3) | 3481 (46.3) | 0.239 |
| Exercise, n (%) | 1698 (67.7) | 5184 (68.8) | 0.311 |

Table S2. Non-significant association of genetic polymorphisms of *HTR2A* gene and risk of hypertension

| Cohort A | |  | | | | | |
| --- | --- | --- | --- | --- | --- | --- | --- |
| SNP |  | Crude | *p* | Model 1 | *p* | Model 2 | *p* |
| rs1360020 | GG | 1.000 | | 1.000 | | 1.000 | |
|  | GA | 0.946 (0.755-1.186) | 0.667 | 0.951 (0.757-1.195) | 0.667 | 1.011 (0.757-1.349) | 0.943 |
|  | AA | 1.229 (0.929-1.626) | 0.137 | 1.239 (0.934-1.643) | 0.137 | 1.294 (0.908-1.843)) | 0.154 |
|  |  |  | |  | |  | |
| rs4941573 | CC | 1.000 | | 1.000 |  | 1.000 |  |
|  | CT | 0.900 (0.714-1.134) | 0.371 | 0.893 (0.707-1.129) | 0.345 | 0.956 (0.711-1.286) | 0.766 |
|  | TT | 1.233 (0.930-1.634 | 0.146 | 1.239 (0.932-1.647 | 0.140 | 1.218 (0.853-1.740) | 0.277 |
|  |  |  | |  | |  | |
| rs6313 | AA | 1.000 | | 1.000 | | 1.000 | |
|  | AG | 0.924 (0.730-1.168) | 0.507 | 0.919 (0.725-1.165) | 0.483 | 0.960 (0.711-1.297) | 0.791 |
|  | GG | 1.193 (0.902-1.579) | 0.217 | 1.195 (0.900-1.586) | 0.217 | 1.233 (0.865-1.758) | 0.247 |
|  |  |  | |  | |  | |
| rs970 | AA | 1.000 | | 1.000 | | 1.000 | |
|  | AC | 0.906 (0.685-1.198) | 0.488 | 0.923 (0.696-1.223) | 0.577 | 0.867 (0.600-1.254) | 0.449 |
|  | CC | 0.573 (0.036-9.181) | 0.694 | 0.656 (0.041-10.526) | 0.766 | 1.390 (0.035-55.901) | 0.861 |
|  |  |  | |  | |  | |
| rs184 | GG | 1.000 | | 1.000 | | 1.000 | |
|  | AG | 1.017 (0.833-1.241) | 0.869 | 1.029 (0.842-1.258) | 0.781 | 1.114 (0.864-1.438) | 0.405 |
|  | AA | 1.262 (0.836-1.905) | 0.268 | 1.228 (0.811-1.860) | 0.331 | 1.113 (0.666-1.860) | 0.682 |
|  |  |  |  |  |  |  |  |
| rs186 | GG | 1.000 |  | 1.000 |  | 1.000 |  |
|  | GT | 1.033 (0.847-1.261) | 0.747 | 1.048 (0.858-1.281) | 0.645 | 1.157 (0.897-1.492) | 0.262 |
|  | TT | 1.367 (0.905-2.067) | 0.137 | 1.328 (0.876-2.013) | 0.182 | 1.179 (0.701-1.980) | 0.535 |
|  |  |  |  |  |  |  |  |
| rs886 | AA | 1.000 |  | 1.000 |  | 1.000 |  |
|  | GA | 1.000 (0.807-1.240) | 0.999 | 1.011 (0.814-1.255) | 0.924 | 1.037 (0.787-1.366) | 0.797 |
|  | GG | 1.185 (0.886-1.585) | 0.252 | 1.198 (0.893-1.607 | 0.228 | 1.148 (0.794-1.659) | 0.464 |
|  |  |  |  |  |  |  |  |
| rs991 | TT | 1.000 |  | 1.000 |  | 1.000 |  |
|  | TC | 0.867 (0.636-1.183) | 0.368 | 1.126 (0.916-1.384) | 0.261 | 1.179 (0.907-1.533) | 0.219 |
|  | CC | 0.966 (0.711-1.311) | 0.822 | 1.152 (0.842-1.576) | 0.377 | 1.083 (0.731-1.605) | 0.692 |

Model 1 was adjusted for age, gender, smoking status, alcohol consumption, and regular exercise. Model 2 was adjusted for Model 1, and additionally adjusted for systolic blood pressure, total cholesterol and baseline body mass index.

Table S3. Non-significant association of genetic polymorphisms of *HTR2A* gene and risk of hypertension

| Cohort B | |  | | | | | |
| --- | --- | --- | --- | --- | --- | --- | --- |
| SNP |  | Crude | *p* | Model 1 | *p* | Model 2 | *p* |
| rs977003 | AA | 1.000 | | 1.000 | | 1.000 | |
|  | AC | 1.023 (0.932-1.123) | 0.627 | 1.011 (0.918-1.115) | 0.820 | 1.032 (0.912-1.169) | 0.615 |
|  | CC | 1.065 (0.879-1.290) | 0.523 | 1.065 (0.871-1.301) | 0.539 | 1.120 (0.871-1.440) | 0.379 |
|  |  |  | |  | |  | |
| rs9316232 | TT | 1.000 | | 1.000 | | 1.000 | |
|  | TC | 0.944 (0.850-1.049) | 0.288 | 0.936 (0.838-1.045) | 0.238 | 0.967 (0.840-1.114) | 0.644 |
|  | CC | 1.005 (0.888-1.138) | 0.933 | 1.008 (0.886-1.147) | 0.906 | 1.035 (0.878-1.221) | 0.680 |
|  |  |  | |  | |  | |
| rs582854 | GG | 1.000 | | 1.000 | | 1.000 | |
|  | GT | 0.961 (0.876-1.054) | 0.397 | 0.970 (0.880-1.068) | 0.530 | 0.931 (0.808-1.072) | 0.321 |
|  | TT | 0.991 (0.835-1.176) | 0.919 | 0.974 (0.815-1.165) | 0.773 | 0.832 (0.638-1.084) | 0.174 |
|  |  |  | |  | |  | |
| rs1360020 | AA | 1.000 | | 1.000 | | 1.000 | |
|  | AC | 1.080 (0.973-1.199) | 0.150 | 1.097 (0.984-1.224) | 0.096 | 1.141 (0.973-1.339) | 0.105 |
|  | CC | 0.947 (0.835-1.074) | 0.397 | 0.955 (0.838-1.089) | 0.489 | 0.855 (0.706-1.036) | 0.109 |
|  |  |  | |  | |  | |
| rs2183057 | AA | 1.000 | | 1.000 | | 1.000 | |
|  | AG | 1.088 (0.988-1.199) | 0.087 | 1.094 (0.989-1.211) | 0.081 | 1.121 (0.967-1.300) | 0.131 |
|  | GG | 0.993 (0.871-1.133) | 0.918 | 0.989 (0.862-1.134) | 0.872 | 0.881 (0.719-1.079) | 0.220 |
|  |  |  |  |  |  |  |  |
| rs9590999 | CC | 1.000 |  | 1.000 |  | 1.000 |  |
|  | CT | 1.076 (0.971-1.193) | 0.163 | 1.082 (0.971-1.205) | 0.152 | 1.130 (0.983-1.299) | 0.805 |
|  | TT | 1.000 (0.882-1.133) | 1.011 | 1.011(0.887-1.152) | 0.871 | 1.046 (0.884-1.239) | 0.599 |

Model 1 was adjusted for age, gender, smoking status, alcohol consumption, and regular exercise. Model 2 was adjusted for Model 1, and additionally adjusted for systolic blood pressure, total cholesterol and baseline body mass index.

Table S4. Distribution of *HTR2A* genetic variations according to development of hypertension

| Cohort A (N=6039) | | | | |  |
| --- | --- | --- | --- | --- | --- |
| SNP | Genotype | No  Hypertension  (N=4621), N (%) | Hypertension  (N=1418), N (%) | *p*-value | adjusted *p*-value* |
| rs7330636 | GG | 555 (85.9) | 989 (89.2) | 0.092 | 0.107 |
|  | GA | 89 (13.8) | 115 (10.4) |  |  |
|  | AA | 2 (0.3) | 5 (0.5) |  |  |
| rs9590999 | CC | 167 (25.1) | 335 (29.2) | 0.056 | 0.087 |
|  | CT | 354 (53.2) | 545 (47.6) |  |  |
|  | TT | 144 (21.7) | 266 (23.2) |  |  |
| rs2183057 | AA | 253 (38.0) | 401 (34.9) | 0.026 | 0.087 |
|  | AG | 331 (49.7) | 553 (48.1) |  |  |
|  | GG | 82 (12.3) | 195 (17.0) |  |  |
| rs4942595 | TT | 168 (25.2) | 339 (29.5) | 0.041 | 0.087 |
|  | TC | 354 (53.1) | 541 (47.1) |  |  |
|  | CC | 145 (21.7) | 269 (23.4) |  |  |
| rs4531630 | AA | 168 (25.3) | 338 (29.6) | 0.062 | 0.087 |
|  | AG | 344 (51.7) | 584 (51.1) |  |  |
|  | GG | 153 (23.0) | 221 (12.2) |  |  |
| rs17069883 | AA | 197 (29.5) | 287 (25.0) | 0.041 | 0.087 |
|  | AC | 341 (51.1) | 594 (51.7) |  |  |
|  | CC | 129 (19.3) | 268 (23.3) |  |  |
| rs4942578 | CC | 258 (38.7) | 431 (37.5) | 0.225 | 0.225 |
|  | CA | 320 (48.0) | 530 (46.1) |  |  |
|  | AA | 89 (13.3) | 188 (16.4) |  |  |

Cohort B (N=7524) (N=4999) (N=2525)

| rs4942578 | CC | 1834 (39.2) | 949 (40.2) | 0.605 | 0.605 |
| --- | --- | --- | --- | --- | --- |
|  | CA | 2171 (46.5) | 1089 (46.2) |  |  |
|  | AA | 668 (14.3) | 320 (13.6) |  |  |
| rs985933 | AA | 1526 (32.8) | 781 (33.2) | 0.496 | 0.595 |
|  | AG | 2257 (48.5) | 1154 (49.1) |  |  |
|  | GG | 875 (18.8) | 414 (17.6) |  |  |
| rs4941573 | TT | 1184 (25.4) | 565 (24.0) | 0.196 | 0.389 |
|  | TC | 2314 (49.6) | 1221 (51.8) |  |  |
|  | CC | 1172 (25.1) | 571 (24.2) |  |  |
| rs6313 | GG | 1229 (26.3) | 584 (24.8) | 0.153 | 0.389 |
|  | GA | 2301 (49.2) | 1218 (51.7) |  |  |
|  | AA | 1143 (24.5) | 556 (23.6) |  |  |
| rs1328685 | TT | 4031 (86.3) | 2054 (87.1) | 0.259 | 0.389 |
|  | TC | 611 (13.1) | 295 (12.5) |  |  |
|  | CC | 31 (0.7) | 9 (0.4) |  |  |
| rs2567675 | TT | 2823 (60.4) | 1483 (62.9) | 0.124 | 0.389 |
|  | TC | 1616 (34.6) | 762 (32.3) |  |  |
|  | CC | 234 (5.0) | 112 (4.8) |  |  |

*, adjusted p-value was calculated with the Benjamini-Hochberg method

Table S5. Association of genetic polymorphisms of *HTR2A* gene and risk of hypertension

| Cohort A | | Crude | | Model 1 | | Model 2 | |
| --- | --- | --- | --- | --- | --- | --- | --- |
| SNP |  | OR (adj. 95% CI)*§* | *p (adj. p)§* | OR (adj. 95% CI)^*^ | *p (adj. p)* | OR (adj. 95% CI)^#^ | *p (adj. p)* |
| rs7330636 | GG | 1.000 | | 1.000 | | 1.000 | |
|  | GA | 0.725 (0.467-1.128) | 0.033 (0.077) | 0.725 (0.463-1.134) | 0.034 (0.079) | 0.644 (0.330-1.255) | 0.026 (0.098) |
|  | AA | 1.403 (0.638-3.087) | 0.686 (0.800) | 1.520 (0.226-10.240) | 0.619 (0.667) | 1.415 (0.451-4.439) | 0.724 (0.724) |
| rs9590999 | CC | 1.000 | | 1.000 |  | 1.000 |  |
|  | CT | 0.767 (0.545-1.080) | 0.023 (0.064) | 0.757 (0.542-1.056) | 0.018 (0.050) | 0.738 (0.465-1.170) | 0.042 (0.098) |
|  | TT | 0.921 (0.829-1.024) | 0.921 (0.936) | 0.914 (0.552-1.512) | 0.522 (0.637) | 0.880 (0.289-2.674) | 0.463 (0.589) |
| rs2183057 | AA | 1.000 | | 1.000 | | 1.000 | |
|  | AG | 1.054 (0.927-1.200) | 0.620 (0.789) | 1.067 (0.742-1.535) | 0.546 (0.637) | 1.131 (0.003-370.600) | 0.369 (0.517) |
|  | GG | 1.500 (0.889-2.534) | 0.009 (0.064) | 1.500 (0.924-2.432) | 0.009 (0.050) | 1.448 (0.797-2.628) | 0.056 (0.112) |
| rs4942595 | TT | 1.000 | | 1.000 | | 1.000 | |
|  | TC | 0.757 (0.529-1.084) | 0.017 (0.064) | 0.748 (0.528-1.058) | 0.013 (0.050) | 0.739 (0.467-1.169) | 0.042 (0.098) |
|  | CC | 0.919 (0.733-1.153) | 0.547 (0.766) | 0.911 (0.542-1.533) | 0.510 (0.637) | 0.907 (0.585-1.404) | 0.574 (0.670) |
| rs4531630 | AA | 1.000 | | 1.000 | | 1.000 | |
|  | AG | 0.844 (0.544-1.309) | 0.144 (0.224) | 0.834 (0.557-1.250) | 0.122 (0.190) | 0.792 (0.459-1.367) | 0.115 (0.201) |
|  | GG | 0.718 (0.469-1.101) | 0.019 (0.064) | 0.705 (0.465-1.070) | 0.015 (0.050) | 0.658 (0.349-1.241) | 0.021 (0.098) |
| rs17069883 | AA | 1.000 |  | 1.000 |  | 1.000 |  |
|  | AC | 1.196 (0.774-1.848) | 0.119 (0.210) | 1.197 (0.801-1.789) | 0.121 (0.190) | 1.197 (0.486-2.944) | 0.224 (0.348) |
|  | CC | 1.426 (0.902-2.255) | 0.012 (0.064) | 1.449 (0.930-2.257) | 0.009 (0.050) | 1.447 (0.827-2.530) | 0.040 (0.098) |
| rs4942578 | CC | 1.000 |  | 1.000 |  | 1.000 |  |
|  | CA | 0.991 (0.980-1.002) | 0.935 (0.936) | 1.007 (0.999-1.015) | 0.950 (0.950) | 1.054 (0.885-1.256) | 0.696 (0.724) |
|  | AA | 1.264 (0.715-2.239) | 0.120 (0.210) | 1.288 (0.732-2.264) | 0.096 (0.190) | 1.562 (0.794-3.071) | 0.020 (0.098) |

OR, odds ratio; 95% CI, 95% confidential interval.

^*^, Model 1 was adjusted for gender, smoking status, alcohol consumption, regular exercise and age.

^#^, Model 2 was adjusted for Model 1 and systolic blood pressure, total cholesterol and baseline body mass index additionally.

*§*, adjusted p-value and 95% CI were corrected with Hochberg step-up method.

Table S6. Association of genetic polymorphisms of *HTR2A* gene and risk of hypertension

| Cohort B | | Crude | | | Model 1 | | | Model 2 | | |
| --- | --- | --- | --- | --- | --- | --- | --- | --- | --- | --- |
| SNP |  | OR (adj. 95% CI)*§* | *P (adj. p)§* | OR (adj. 95% CI)^*^ | | *P (adj. p)* | OR (adj. 95% CI)^#^ | |  | *P (adj. p)* |
| rs4942578 | CC | 1.000 | | 1.000 | | | 1.000 | | | |
|  | CA | 0.969 (0.889-1.057) | 0.569 (0.759) | 0.979 (0.927-1.034) | | 0.710 (0.775) | 0.894 (0.576-1.387) | | | 0.180 (0.309) |
|  | AA | 0.926 (0.636-1.348) | 0.328 (0.656) | 0.949 (0.826-1.088) | | 0.521 (0.775) | 0.735 (0.521-1.038) | | | 0.010 (0.040) |
| rs985933 | AA | 1.000 | | 1.000 | | | 1.000 | | | |
|  | AG | 0.999 (0.998-1.000) | 0.986 (0.986) | 1.013 (0.986-1.041) | | 0.824 (0.824) | 1.057 (0.559-2.000) | | | 0.521 (0.568) |
|  | GG | 0.924 (0.628-1.359) | 0.289 (0.656) | 0.922 (0.522-1.628) | | 0.293 (0.610) | 0.789 (0.572-1.088) | | | 0.037 (0.074) |
| rs4941573 | TT | 1.000 | | 1.000 | | | 1.000 | | | |
|  | TC | 1.106 (0.445-2.751) | 0.106 (0.414) | 1.137 (0.851-1.516) | | 0.048 (0.192) | 1.327 (1.019-1.729) | | | 0.003 (0.018) |
|  | CC | 1.021 (0.981-1.064) | 0.774 (0.844) | 1.033 (0.950-1.122) | | 0.670 (0.775) | 1.113 (0.493-2.513) | | | 0.332 (0.398) |
| rs6313 | GG | 1.000 | | 1.000 | | | 1.000 | | | |
|  | GA | 1.114 (0.421-2.951) | 0.079 (0.414) | 1.157 (0.890-1.505) | | 0.023 (0.138) | 1.365 (1.042-1.788) | | | 0.001 (0.012) |
|  | AA | 1.024 (0.979-1.070) | 0.745 (0.844) | 1.042 (0.937-1.159) | | 0.586 (0.775) | 1.130 (0.589-2.173) | | | 0.267 (0.356) |
| rs1328685 | TT | 1.000 | | 1.000 | | | 1.000 | | | |
|  | TC | 0.948 (0.788-1.139) | 0.478 (0.717) | 0.939 (0.769-1.146) | | 0.427 (0.732) | 0.982 (0.952-1.013) | | | 0.875 (0.875) |
|  | CC | 0.570 (0.004-91.460) | 0.138 (0.414) | 0.544 (0.018-16.396) | | 0.121 (0.363) | 0.292 (0.057-1.492) | | | 0.029 (0.070) |
| rs2567675 | TT | 1.000 |  | 1.000 | |  | 1.000 | | |  |
|  | TC | 0.898 (0.339-2.378) | 0.047 (0.414) | 0.868 (0.672-1.120) | | 0.012 (0.138) | 0.832 (0.653-1.061) | | | 0.027 (0.070) |
|  | CC | 0.911 (0.663-1.252) | 0.435 (0.717) | 0.880 (0.361-2.147) | | 0.305 (0.610) | 0.799 (0.311-2.055) | | | 0.214 (0.321) |

OR, odds ratio; 95% CI, 95% confidential interval.

^*^, Model 1 was adjusted for gender, smoking status, alcohol consumption, regular exercise and age.

^#^, Model 2 was adjusted for Model 1 and systolic blood pressure, total cholesterol and baseline body mass index additionally.

*§*, adjusted p-value and 95% CI were corrected with Hochberg step-up method.
